# Prognostic Significance of the Combined Score of Endothelial Expression of Nucleolin and CD31 in Surgically Resected Non-Small Cell Lung Cancer

**DOI:** 10.1371/journal.pone.0054674

**Published:** 2013-01-31

**Authors:** Hongyun Zhao, Yan Huang, Cong Xue, Yang Chen, Xue Hou, Ying Guo, Liping Zhao, Zhi huang Hu, Yujie Huang, Yongzhang Luo, Li Zhang

**Affiliations:** 1 State Key Laboratory of Oncology in South China, Cancer Center, Sun Yat-Sen University, Guangzhou, China; 2 National Engineering Laboratory for Anti-tumor Protein Therapeutics, Beijing Key Laboratory for Protein Therapeutics, and Cancer Biology Laboratory, School of Life Sciences, Tsinghua University, Beijing, China; Okayama University, Japan

## Abstract

Nucleolin is implicated to play a role in angiogenesis, a vital process in tumor growth and metastasis. However, the presence and clinical relevance of nucleolin in human non small cell lung cancer (NSCLC) remains largely unknown. In this study, we explored the expression and prognostic implication of nucleolin in surgically resected NSCLC patients. A cohort of 146 NSCLC patients who underwent surgical resection was selected for tissue microarray. In this tissue microarray, nucleolin expression was measured by immunofluorescence. Staining for CD31, a marker of endothelial cells, was performed to mark blood vessels. A Cox proportional hazards model was used to assess the prognostic significance of nucleolin. Nucleolin expression was observed in 34.2% of all patients, and 64.1% in high CD31 expression patients. The disease-free survival (DFS) was significantly shorter in patients with high nucleolin (CD31^hi^NCL^hi^) compared to patients with low tumor blood vessels (CD31^lo^NCL^lo^) (5 ys of DFS 24% vs 64%, *p* = 0.002). Such a difference was demonstrated in the following stratified analyses: stage I (*p*<0.001), squamous cell carcinoma and adenosquamous cell carcinoma (*p* = 0.028), small tumor (<5 cm, *p* = 0.008), and surgery alone (*p* = 0.015). Multivariate analysis further revealed that nucleolin expression independently predicted for worse survival (*p* = 0.003). This study demonstrates that nucleolin is associated with the clinical outcomes in postoperative NSCLC patients. Thus, the expression levels of nucleolin may provide a new prognostic marker to identify patients at higher risk for treatment failure, especially in some subgroups.

## Introduction

Lung cancer is the leading cause of cancer deaths in men and women [1]. Non–small cell lung cancer (NSCLC) accounts for approximately 85% of all lung cancer, and it is typically associated with poor outcomes. The recurrence rate of NSCLC is considerably high in patients with complete resection of the primary tumor, which accounts for the highest cancer-related mortality worldwide. For stage I/II NSCLC, the relapse rate is around 35–67% and the 5-year survival rate ranges from 33–63% [2], [3]. In fact, most treatment failures are largely due to distant recurrences.

Angiogenesis is a critical process during tumor development and metastasis [4], [5]. It has been demonstrated that neoangiogenesis is a significant prognostic factor for both overall survival and disease free survival in lung cancer [6]–[12]. Numerous studies have measured tumor microvessel density (MVD) by CD31 staining. However, the association between MVD and survival in patients with lung cancer remains unresolved [13].

Nucleolin is a ubiquitous non-histone protein that was initially isolated from the nucleolus [14]. As a multifunctional protein, nucleolin plays fundamental roles in many important processes, including cell proliferation, ribosome assembly, rDNA transcription, packaging of pre-RNA, and organization of nucleolar chromatin [15], [16]. However, accumulating evidence indicates that nucleolin can shuttle to the cytoplasm and membrane from the nucleolus [17]–[20]. Although nucleolin is predominantly localized in the nucleolus, it has also been found at the cell surface of various cell types in a phosphorylated form [21]. Recently, nucleolin expression was observed on the surface of endothelial cells in nascent tumor blood vessels [22], [23]. In the absence of cell surface nucleolin, the endothelial cells partially lost their motility and failed to form tubule structures [24]. Moreover, as a novel functional receptor for endostain, nucleolin is responsible for the potent antiangiogenic and antitumor activities of endostain [25], [26]. Previous studies have found nucleolin expression localized to most blood vessels of xenograft tumors, suggesting the potential involvement of nucleolin in tumor angiogenesis [23], [26]. However, so far, there are currently few reports which study the effects of nucleolin in human NSCLC.

Based on the previous studies of prognostic biomarkers in NSCLC patients [27], [28] and the involvement of nucleolin in tumor angiogenesis, we hypothesized that nucleolin may serve as an important marker for tumor neoangiogenesis and is associated with a poor prognosis in early NSCLC after radical surgery. In this study, we examined the relation between nucleolin expression and clinicopathogical variables in 146 operable NSCLC patients.

## Materials and Methods

### Patients

A total of 146 consecutive NSCLC patients treated with radical surgery from August 2000 to November 2004 at the Cancer Center of Sun Yat-Sen University were enrolled in this study. The study was approved by the Medical Ethics Committee of Sun Yat-Sen University. Patients with previous history of malignant diseases were excluded. Tumor tissue samples and follow-up data were available for all included cases. Patient demographics are listed in Table 1. The grade of tumor differentiation was determined according to the criteria of the World Health Organization. The pathologic Tumor-Node-Metastasis (TNM) status was assessed by the TNM classification of the International Union against Cancer (UICC, 2002). Disease-free survival was defined as the period from the date of surgery to the date of disease progression or death. If no disease progression or death was observed at the end of follow-up, disease-free survival was defined from the date of surgery to the last time of tumor evaluation. Overall survival was defined as the period from the date of surgery to the date of death or the last follow-up. Bone metastasis time was defined from the date of surgery to the date of bone metastasis. If no bone metastasis was noted in the last follow-up, bone metastasis time was calculated as the interval from the date of surgery to the last time of tumor evaluation. The starting date of neoadjuvant chemotherapy was ignored. The end date of the follow-up study was March 3, 2010. The median follow-up was 70.8 months (range: 10.4–114 months).

**Table 1:** Clinicopathologic variables as prognostic predictors for disease-free survival (DFS) and overall survival (OS) in 146 NSCLC patients (Univariate analyses; Log-rank test).

Characteristic	No. of patients	5-year DFS		5-year OS	
		%	*P* value	%	*P* value
**Gender**					
Male	110	48		69	
Female	36	46	0.943	69	0.891
**Age**					
<60 y	77	43		7	
≥60 y	69	52	0.358	68	0.589
**Histology**					
ADC*	86	43		69	
SCC‡	52	54		70	
ADSCC§	8	51	0.703	62	0.595
**Cancer differentiation**					
Low	64	34		59	
Median, High	82	57	**0.036**	76	**0.038**
**Tumor size**					
<5 cm	92	55		78	
≥5 cm	54	33	**0.004**	54	**0.001**
**N stage**					
0	81	62		84	
1	26	50		66	
2	38	15		39	
3	1	0	**<0.001**	0	**<0.001**
**Pathologic stage**					
I	64	68		91	
II	35	47		61	
III	47	19	**<0.001**	44	**<0.001**
**Treatment**					
Surgery	86	53		73	
Surgery/Chemotherapy	44	45		68	
Surgery/Radiation	16	23	**0.012**	50	0.142

* ADC = adenocarcinoma,

‡ SCC = squamous cell carcinoma,

§ ADSCC = adenosquamous cell carcinoma.

### Construction of Tissue Microarrays (TMA)

The TMA was constructed according to a method previously described [29]. Briefly, the individual donor tissue block and the corresponding hematoxylin and eosin (H&E)-stained slides were overlaid for tissue TMA sampling. Tissue was sampled using a tissue arraying instrument (Beecher Instruments, Silver Spring, MD, USA). A 0.6-mm diameter core of tissues was removed and re-embedded into a predetermined position in a recipient paraffin block. Three core samples were selected from each primary tumor. Multiple sections (5 µm thick) were cut from TMA block and mounted on adhesive-coated slides using the Paraffin Tape-Transfer System (Instrumedics, Hackensack, NJ, USA).

### Immunohistochemistry

According to methods previously described [23], [26], the deparaffinized sections were incubated at 4°C overnight with mouse anti-human CD31 mAb (Santa Cruz Biotechnologies, Santa Cruz, CA, USA) and rabbit anti-human nucleolin pAb (Protgen, Beijing, China). After rinsing with TBS-T (0.1% Tween 20 in TBS), samples were incubated with FITC-conjugated goat anti-mouse and TRITC-conjugated goat anti-rabbit secondary antibodies (Jackson Immuno-Research Laboratories, West Grove, PA, USA) for 1 hour at room temperature. The nuclei were stained with DAPI. Confocal fluorescence images were captured from at least 4 random fields per section at 100×magnification using a Nikon A1 laser scanning confocal imaging system (Nikon, Japan). Images were obtained and evaluated with multiple photomultiplier tubes regulated by Nikon NIS-elements software. All slides were reviewed by two independent pathologists who were blinded to the clinical and pathological information.

### Statistical Analysis

SPSS16.0 software (SPSS Inc, Chicago, IL, USA) was used for data analysis.

Disease-free survival and overall survival were estimated using the Kaplan-Meier method and analyzed using the log-rank test. The association of nucleolin expression with clinical and pathological characteristics was assessed using the Chi-square test. Statistically significant variables were analyzed using a univariate Cox proportional hazards model. Multivariate survival analysis was performed on all parameters to test for independent prognostic values. Results were considered significant at P<0.05.

## Results

### Cohort Description and Clinical Predictors

The clinical and pathologic characteristics of each patient are listed in Table 1. The mean tumor size was 4.25 cm (range, 1–12 cm). Sixty of 146 patients (41%) who underwent surgery also received chemotherapy or radiation therapy. Tumor progression was noted in 71 patients (49%), with distant recurrence in 62 patients and local recurrence in 9 patients. Fifty-three patients (36%) died during the follow-up time. The estimated 5-year disease-free survival rate was 47% (95% CI, 40.7%–59.3%), and the 5-year overall survival rate was 69% (95% CI, 63.2%–78.7%) for all 146 patients. As expected, the univariate analysis revealed significant associations of cancer differentiation, tumor size, N stage, and pathologic stage with both disease-free survival and overall survival. However, the treatment modality was only associated with disease-free survival but not overall survival (Table 1).

### Nucleolin Expression in NSCLC

Nucleolin was detected in 50 patients out of the total patient cohort (34.2%). Further analysis with pathological factors revealed that nucleolin expression was significantly associated with large tumors (≥5 cm) (large versus small: 44.4% vs 28.3%, *P* = 0.047). There were no significant relationships between nucleolin and other factors, such as age, gender, histological subtype, cancer differentiation and pathologic stages.

**Figure 1:**
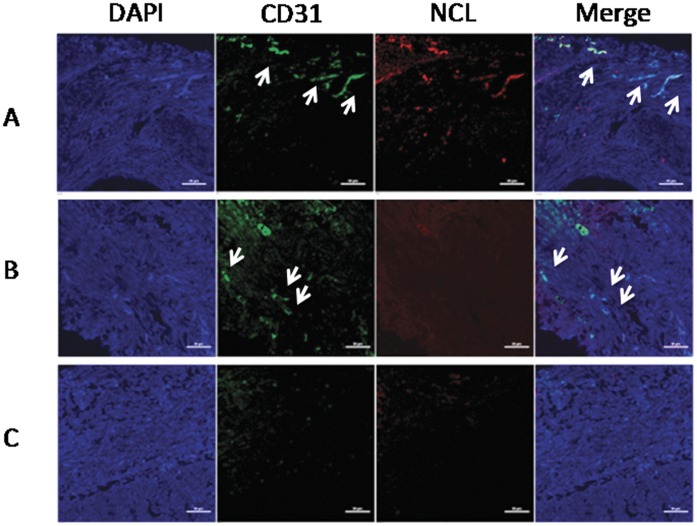
The NSCLC sections were divided into three groups based on the blood vessel density and nucleolin expression. The tumor blood vessels and nucleolin were stained with anti-CD31 (green) and anti-nucleolin (red), respectively. Nuclei were stained with DAPI (blue). Representative images from the three groups were shown here. (A) CD31^hi^NCL^hi^, (B) CD31^hi^NCL^lo^, (C) CD31^lo^NCL^lo^. Scale bar, 50 µm.

Notably, high CD31 expression was noted in 53.2% of the entire cohort (n = 78). Nucleolin was colocalized with the pan-endothelial marker CD31 in all 50 patients with nucleolin expression (64.1%). However, nucleolin was absent in other 28 patients with CD31 expression. These findings suggest a potential role for nucleolin in the development of tumor angiogenesis. The patients were further divided into three groups based on the status of nucleolin and CD31 expression (Figure 1). This included high occurrence of tumor blood vessels with nucleolin expression (CD31^hi^NCL^hi^), an average blood vessel number of more than 10/field, and typical blood vessel morphology should be apparent. Furthermore, expression level of NCL on those tumor blood vessels is significantly higher (at least 2-fold greater mean fluorescence intensity, measured by Nikon NIS-Elements Software) than basal NCL level within the entire tumor region. Increased co-localization was also observed between CD31 positive tubular structures and NCL positive areas. Patients were also further divided by the high occurrence of tumor blood vessels without nucleolin expression (CD31^hi^NCL^lo^). These criteria include a similar high blood vessel density as those of the CD31^hi^NCL^hi^ group, expression level of NCL on tumor blood vessels was not significantly higher (<2-fold, mean fluorescence intensity) than the basal NCL level within the entire tumor tissue. Moreover, no significant differences were observed between the co-localization of CD31 positive tubular structures and NCL positive areas.

In terms of low tumor blood vessels (CD31^lo^NCL^lo^), the average number of blood vessel with typical tubular structure should be less than 10/field. These tumor blood vessels should neither express high nucleolin nor co-localize with CD31 positive vessels. Hardly any low blood vessel density-high nucleolin expression section was observed among the NSCLC sections.

### Univariate Survival Analysis

**Figure 2:**
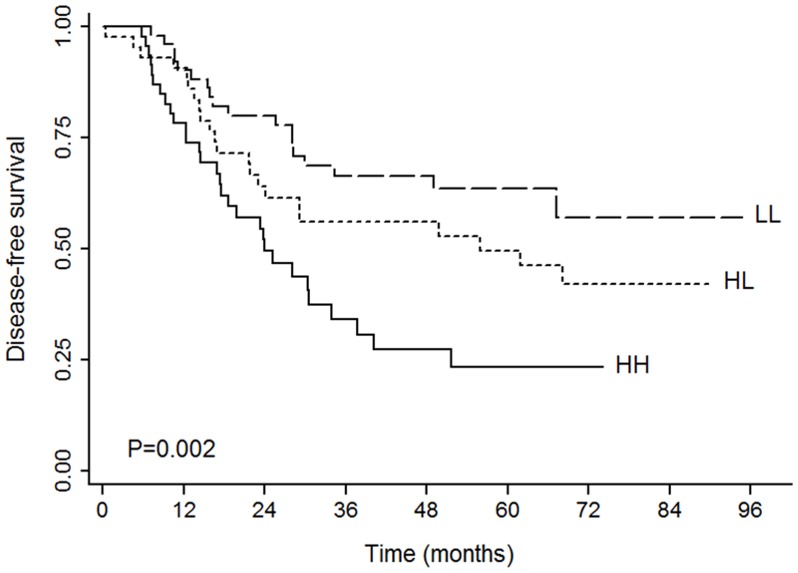
Kaplan-Meier plots of DFS according to the expression of CD31 and nucleolin in the entire cohort. HH: CD31^hi^NCL^hi^, HL: CD31^hi^NCL^lo^, and LL: CD31^lo^NCL^lo^.

**Figure 3:**
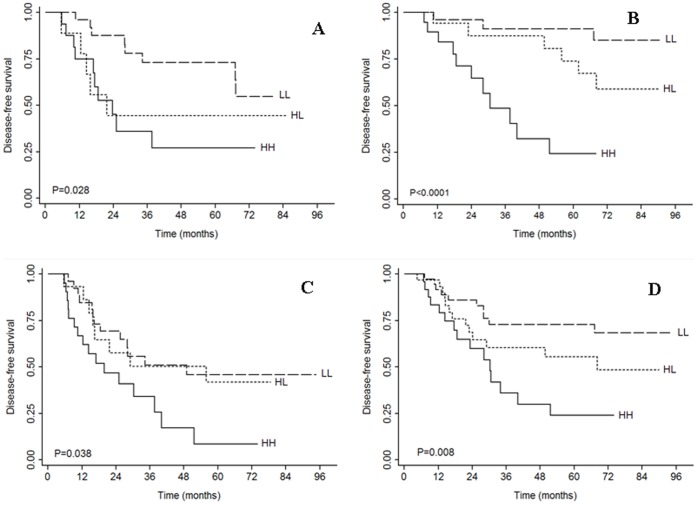
Kaplan-Meier plots of DFS according to the expression of CD31 and nucleolin in the subgroups: (A) Squamous cell carcinoma (SCC) and adenosquamous cell carcinoma (ADSCC), (B) Tumor size <5 cm, (C) Stage I, and (D) Surgery alone. HH: CD31hiNCLhi, HL: CD31hiNCLlo, and LL: CD31loNCLlo.

We used univariate analysis to determine the impact of nucleolin expression on the survival of NSCLC patients with resectable tumors. The survival data is presented in Table 2. Nucleolin expression was significantly associated with decreased disease-free survival (*P = *0.002, figure 2) but did not impact overall survival (*P = *0.841). Stratified analysis revealed a significant association of nucleolin expression with the disease-free survival in patients with squamous or adenosquamous cell carcinoma (*P = *0.028, figure 3) but not in patients with adenocarcinoma (*P = *0.061). Nucleolin expression was also associated with disease-free survival when stratified by other tumor characteristics, such as low cancer differentiation (*P = *0.038, figure 3), median/high cancer differentiation (*P = *0.01), surgical treatment alone (*P = *0.015), and smaller tumor size (*P = *0.008, figure 3). More importantly, nucleolin had a significant impact on disease-free survival in stage I patients (*P*<0.0001, figure 3) but not in patients with stage II to IIIA disease (*P = *0.374). However, there were no significant differences for overall survival among the three nucleolin groups in relation to clinical and pathologic tumor variables.

**Table 2:** Univariate analyses of DFS and OS according to the expression of CD31 and nucleolin in all patients and subgroups with clinicopathologic variables (Log-rank test).

Surface expression	No. of patients (%)	DFS (5-year)		OS (5-year)	
		(%)	*P* value	(%)	*P* value
**Total**					
CD31^lo^NCL^lo^	52 (35.7)	64		72	
CD31^hi^NCL^lo^	44 (30.1)	50		65	
CD31^hi^NCL^hi^	50 (34.2)	24	**0.002**	69	0.841
**Histology**					
ADC*					
CD31^lo^NCL^lo^	25 (29.1)	57		71	
CD31^hi^NCL^lo^	31 (36.0)	47		62	
CD31^hi^NCL^hi^	30 (34.9)	21	0.061	73	0.837
SCC‡+ADSCC§					
CD31^lo^NCL^lo^	27 (45)	70		73	
CD31^hi^NCL^lo^	13 (21.7)	56		69	
CD31^hi^NCL^hi^	20 (33.3)	27	**0.028**	64	0.805
**Tumor differentiation**					
Low					
CD31^lo^NCL^lo^	26 (40.6)	46		64	
CD31^hi^NCL^lo^	16 (25)	42		56	
CD31^hi^NCL^hi^	22 (34.4)	8	**0.038**	57	0.782
Median, High					
CD31^lo^NCL^lo^	26 (31.8)	82		80	
CD31^hi^NCL^lo^	28 (34.1)	54		70	
CD31^hi^NCL^hi^	28 (34.1)	35	**0.01**	79	0.873
**Tumor size**					
<5 cm					
CD31^lo^NCL^lo^	36 (39.1)	73		74	
CD31^hi^NCL^lo^	30 (32.6)	56		76	
CD31^hi^NCL^hi^	26 (28.3)	24	**0.008**	85	0.843
≥5 cm					
CD31^lo^NCL^lo^	16 (29.6)	43		69	
CD31^hi^NCL^lo^	14 (25.9)	37		39	
CD31^hi^NCL^hi^	24 (44.5)	26	0.392	51	0.795
**Overall stage**					
Stage I					
CD31^lo^NCL^lo^	26 (40.6)	91		88	
CD31^hi^NCL^lo^	17 (26.6)	74		94	
CD31^hi^NCL^hi^	21 (32.8)	25	**<0.001**	90	0.474
Stage II∼III					
CD31^lo^NCL^lo^	26 (31.7)	37		55	
CD31^hi^NCL^lo^	27 (32.9)	35		45	
CD31^hi^NCL^hi^	29 (35.4)	24	0.374	54	0.825
**Treatment**					
Surgery alone					
CD31^lo^NCL^lo^	34 (39.5)	69		73	
CD31^hi^NCL^lo^	20 (23.3)	61		67	
CD31^hi^NCL^hi^	32 (37.2)	29	**0.015**	78	0.969
Surgery+other(s)					
CD31^lo^NCL^lo^	18 (30)	56		71	
CD31^hi^NCL^lo^	24 (40)	39		63	
CD31^hi^NCL^hi^	18 (30)	14	0.088	56	0.619

Uniltivariate hazards ratio analysis was used to assess the significant association between nucleolin and disease-free survival. Overall, patients with nucleolin expression (CD31^hi^NCL^hi^) had a higher risk of recurrence compared to patients with low tumor vessels (CD31^lo^NCL^lo^) (HR = 2.768, 95% CI 1.544∼4.964, *P* = 0.001). When compared to the CD31^hi^NCL^hi^ group, the CD31^hi^NCL^lo^ group had a lower risk of recurrence (HR = 0.568, 95% CI 0.325∼0.991, *P* = 0.046). Further analysis showed that the CD31^hi^NCL^hi^ group also had a highest risk of recurrence in the following subgroups: squamous cell carcinoma and adenosquamous cell carcinoma (HR = 3.907, 95% CI 1.285∼7.462, *P = *0.012), low and median/high differentiation (HR = 2.363, 95%CI 1.129∼4.949, *P = *0.023 and HR = 4.201, 95%CI 1.568∼11.253, *P = *0.004, respectively), small tumor (<5 cm) (HR = 3.382, 95%CI 1.505∼7.603, *P* = 0.003), stage I (HR = 11.371, 95%CI 3.003∼43.059, *P*<0.001), and surgery alone (HR = 3.012, 95% CI 1.345∼6.747, *P = *0.007). In these subgroup analyses, compared to the CD31^lo^NCL^lo^ group, the CD31^hi^NCL^lo^ group was not associated with a higher risk of recurrence except in patients with median or high cancer differentiation (HR = 2.826, 95% CI 1.081∼7.386, *P* = 0.034). Thus, nucleolin may become a valuable marker to identify the risk of recurrence for patients with early stage NSCLC.

### Multivariate Hazards Ratio Analysis

A multivariate survival analysis using Cox’s proportional hazard model for the selection of the prognostic factors for disease-free survival, including clinicopathological parameters mentioned above and immunofluorescence costaining results of the blood vessel density and nucleolin expression level, indicated that both nucleolin expression on tumor vessels and clinical stage III are independent factors of poor prognosis for disease-free survival (*P* = 0.003 and *P*<0.0001, respectively,Table 3). However, advanced stage but not nucleolin expression was an independent prognostic factor for overall survival (*P*<0.001 for stage II/III).

**Table 3:** Multivariate Cox’s proportional hazards model analyses of survival and bone metastasis (BMT).

	HR	95% CI	*P*-value
**DFS**			
Nucleolin			
CD31^lo^NCL^lo^	1.00		
CD31^hi^NCL^lo^	1.462	0.790∼2.706	0.226
CD31^hi^NCL^hi^	2.414	1.346∼4.331	**0.003**
Stage			
Stage I	1.00		
Stage III	4.265	2.420∼7.515	**<0.001**
**OS**			
Stage			
Stage I	1.00		
Stage II	4.312	1.919∼9.688	**<0.001**
Stage III	6.755	3.162∼14.431	**<0.001**
**BMT**			
Nucleolin			
CD31^lo^NCL^lo^	1.00		
CD31^hi^NCL^lo^	1.342	0.503∼3.579	0.556
CD31^hi^NCL^hi^	1.578	0.621∼4.009	0.338
Stage			
Stage I	1.00		
Stage II	5.292	1.396∼20.055	**0.014**
Stage III	9.497	2.727∼33.066	**<0.001**

Bone metastasis is a common complication of NSCLC. The 5-year survival rates of patients’ with bone metastasis, and CD31^hi^NCL^hi^, CD31^hi^NCL^lo^, CD3l^lo^NC^lo^ are72%, 81%, and 82%, respectively. However, neither CD31^hi^NCL^hi^ nor CD31^hi^NCL^lo^ groups displayed a higher risk for bone metastasis compared to CD31^lo^NCL^lo^ (*P* = 0.556 and *P* = 0.338, respectively). High risk for bone metastasis was only observed in patients with advanced stage NSCLC (*P* = 0.014)(Data not shown).

## Discussion

In this study, we found that nucleolin expression was detected in 34.2% of NSCLC patients with resected tumors. This might be associated with the co-localization of nucleolin and CD31, which is consistent with previous observations of nucleolin on tumor endothelial cells [23]. Nucleolin is also conditionally expressed on the surface of endothelial cells only during tumor angiogenesis [23]. One possibility for the heterogeneity of nucleolin expression on the cell surface is the local variation of endothelial cell proliferation in angiogenic lesions in vivo [24]. Moreover, vascular endothelial growth factor (VEGF), extracellular matrix, and intracellular motor protein are essential for expression of nucleolin on the surface of endothelial cells during angiogenesis [24]. These studies might explain why nucleolin is not easily detectable in tumor tissues lacking CD31 expression in our study. However, studies on the relationship of MVD and nucleolin expression are few, particularly in non small cell lung cancer. The sample size was not large enough to detect patients with low CD31 and high nucleolin, and a larger sample size is needed to confirm this relationship. Further research with more sensitive blood vessel markers is needed. Furthermore, we observed that a subgroup of NSCLC patients expressed CD31 in the absence of nucleolin. To determine whether CD31 alone might serve as a prognostic marker in NSCLC, we divided the patients into three groups: CD31^hi^NCL^hi^, CD31^hi^NCL^lo^ and CD31^lo^NCL^lo^_._

We found that nucleolin expression was only associated with large tumors (≥ 5cm) but not with other pathological factors such as gender, age, or histological grade. One explanation might be that larger tumors have sufficient oxygen and nutrition supplied by tumor angiogenesis. Notably, nucleolin has been shown to be positively correlated with the tumor growth rate [24]. In several recent studies, specific antagonists of nucleolin exhibited anti-tumor activity by inhibiting tumor angiogenesis [23], [26], [30], [31].

This is the first study to investigate the prognostic value of nucleolin expression in NSCLC patients. In multivariate Cox regression analysis, nucleolin expression was evaluated as an independent factor of disease free survival in NSCLC patients (HR = 2.414), suggesting that nucleolin could play an important role in tumor relapse process of NSCLC. Angiogenesis is an essential event for tumor invasion and metastasis. Following tumor removal, the pro-angiogenic environment was able to trigger dormant tumors to rapidly grow [32]–[34]. This is likely due to increased expression of vascular endothelial growth factor (VEGF) or decreased expression of endostatin and angiostatin post surgery, which may alter the pro-angiogenic switch [34], [35]. The enhanced angiogenesis together with the entry of more tumor cells into circulation thus facilitates tumor metastasis [6], [36], [37]. It has been well documented that the appearance of neoangiogenesis is a significant indicator of poor prognosis for both disease-free survival and overall survival in lung cancer [6], [7], [9], [11], [38], [39]. In our study, the major reason for relapse is the distant metastasis (87.3%) that is closely related to angiogenesis. On the other hand, we failed to find that nucleolin is a prognostic factor of overall survival. One possible explanation is that patients would receive different treatment strategies (such as endostain, bevacizumab) after disease progression, which could affect survival prognosis.

In further survival analyses, we found that nucleolin expression was significantly associated with a decreased disease-free survival and a high recurrence risk, especially in the subgroups with stage I, smaller tumor (<5 cm) and surgery alone. Clinically, a small fraction of neoplasm are quite aggressive even at early stages which could be fatal [40]. In some cases, the removal of the primary tumor could accelerate tumor recurrence locally or systemically [34], [41], [42]. Therefore, nucleolin may serve as useful marker to identify patients with a poor outcome and to guide postoperative treatment in early-stage NSCLC.

Bone metastasis is a frequent complication of lung cancer. Experimental observations suggest that bone metastatic tumor cells have the capacity to induce endothelial cell proliferation, migration as well as differentiation [43]. However, in this study, we failed to identify an association of nucleolin expression with a high risk of bone metastasis. A larger cohort may be helpful to further confirm the relationship of nucleolin expression and bone metastasis in NSCLC.

In conclusion, we evaluate for the first time the expression of nucleolin in NSCLC. Our findings provide evidence that the nucleolin is localized with CD31 in tumor tissue of NSCLC, suggesting nucleolin is also found on the surface of tumor endothelial cells. We also demonstrate that nucleolin expression could be use as a negative prognostic factor of disease free survival for NSCLC patients treated with radical surgery. The prognostic indicator of nucleolin could also be used especially in patients with SCC+ADSCC, stage I, small tumor, and surgery alone, indicating that the administration of perioperative anti-NL drugs, such as endostatin, might be useful.
